# Implications of Mutant SOD1 on RNA Processing and Interferon Responses in Amyotrophic Lateral Sclerosis: Omics Data Analysis

**DOI:** 10.7759/cureus.81045

**Published:** 2025-03-23

**Authors:** Naoto Honda, Yasuhiro Watanabe, Hiroki Honda, Mika Uemoto, Hayate Fukuhara, Ritsuko Hanajima

**Affiliations:** 1 Department of Neurology, Tottori University, Yonago, JPN

**Keywords:** amyotrophic lateral sclerosis, cu/zn superoxide dismutase, mrna processing, tar dna-binding protein of 43, type 1 interferon response

## Abstract

Introduction: Cytoplasmic inclusions are observed in motor neurons in amyotrophic lateral sclerosis (ALS) associated with the Cu/Zn superoxide dismutase mutation (mtSOD1). Although these inclusions are a hallmark of the disorder, degeneration is not necessarily initiated in the cytoplasm, nor are these structures the culprit of ALS. The nucleus stores genetic material and acts as the cell’s control center, and a small fraction of mtSOD1 is reported to be distributed in the nucleus. We hypothesized that mtSOD1 in the nucleus contributes to motor neuron degeneration.

Methods: We explored the roles of mtSOD1 in relation to nuclear proteins, chromosomal DNA, and mRNA expression. An immortalized cell line derived from a transgenic ALS mouse model expressing mtSOD1-L126delTT with a FLAG was used for stable immunoprecipitation of mtSOD1-binding molecules using shotgun proteomics and chromatin immunoprecipitation-sequencing (ChIP-seq). We also examined mRNA expression by silencing whole SOD1 (innate mouse Sod1 and mtSOD1) or mtSOD1 alone and compared these patterns against those in non-silenced counterparts.

Results: We identified 392 mtSOD1-interacting proteins in the nucleus. Gene ontology (GO) revealed these proteins to be enriched for “mRNA processing.” Notably, more than 11% of mtSOD1-interacting proteins were expressed concurrently with previously reported wild-type TAR DNA-binding protein 43 (TDP-43)-interacting proteins. ChIP-seq revealed that mtSOD1-interacting DNA portions showed a preference for zinc finger protein-binding motifs. GO analysis of the ChIP-seq data revealed that “mRNA processing” was again enriched among the genes harboring mtSOD1-binding domains. RNA expression analyses revealed that the presence of mouse Sod1 and mtSOD1 induced the overexpression of molecules related to “type 1 IFN responses.”

Conclusions: We revealed that mtSOD1 interacted with nuclear proteins and specific DNA segments and that RNA expression was notably altered when mouse Sod1 and mtSOD1 were silenced. These interactions could play a pivotal role in motor neuron degeneration.

## Introduction

Protein aggregates are common pathological findings in several neurodegenerative diseases, including intracytoplasmic Lewy bodies composed of alpha-synuclein (α-SYN) in Parkinson’s disease (PD) [1], extracellular amyloid β (Aβ) consisting of senile plaques, and intracytoplasmic tau in Alzheimer’s disease (AD) [2]. They are distinct neuropathological markers; however, it is yet to be determined whether these aggregates are a direct cause of neuronal death or a collateral phenomenon accompanying disease progression. It could be that aggregate formation is a survival strategy of debilitating neurons, as these aggregates are only observed in remaining neurons, and consummated inclusions might be less harmful [3]. In contrast, if these aggregates do indeed cause neuronal degeneration, the underlying toxic mechanisms remain elusive. Various explanations for the presence of these aggregates have been proposed, such as dysfunctional energy metabolism, failure of protein quality control, RNA and DNA damage, axonal transport and synaptic dysfunctions, and neural inflammation related to microglia and astrocytes [4]. However, pathogenic processes initiated by the presence of toxic aggregates have not been elucidated.

In the present study, we focused on the nucleus of a familial form of amyotrophic lateral sclerosis (ALS) associated with a Cu/Zn superoxide dismutase mutation (mtSOD1) (ALS1). In this familial form of ALS (FALS), Lewy body-like hyaline inclusions composed of mtSOD1 are observed in the cytoplasm. The toxicity of mtSOD1 has been extensively explored, including its effects on mitochondria, axonal transport, and protein quality control systems [5]. To date, however, only a few researchers have examined the physiological [6-10] and pathological [6,7] roles of SOD1 in the nucleus. Nuclear accumulation of mtSOD1 is observed in SH-SY5Y human neuroblastoma cells transfected to achieve low-level expression of mtSOD1-G93A [6], as well as in transgenic ALS model mice expressing high copy numbers of mtSOD1-G93A, reduced copy numbers of mtSOD1-G93A, and low expression levels of mtSOD1-G37R [7]. Other than SOD1, major players of neurodegeneration are reported to exist in the nucleus. For example, α-SYN, named after the presynaptic and nuclear protein [11], is abundant in the nucleus [1]. α-SYN has been shown to directly bind to DNA, RNA-interacting proteins, and histones, as well as speculated to regulate gene expression [1]. A specific Aβ42-binding domain has been identified in DNA, including domains that regulate the expression of amyloid precursor protein and apolipoprotein E [2]. Tau protein has also been reported to play a role in the nucleus [2]. Furthermore, there are several disorders in which neurodegeneration can be explained solely by the accumulation of pathogenic proteins in the nucleus. For example, in spinal bulbar muscular atrophy, an adult-onset motor neuron disease, androgen receptors with expanded triplet repeats enter the nucleus after binding with testosterone hormone to initiate motor neuron degeneration [12]. In addition, nuclear accumulation of huntingtin has been proposed to cause Huntington’s disease [13].

These insights led us to hypothesize that a fraction of mtSOD1 is present in the nucleus and interacts with nuclear proteins and chromosomal DNA; the presence of mtSOD1 alters the mRNA expression profile; and mtSOD1 in the nucleus contributes to neurodegeneration in ALS. To evaluate these hypotheses, we explored interactions of mtSOD1 with nuclear proteins and chromosomal DNA using proteomics and sequencing technology. Further, we examined how the presence of mtSOD1 changed mRNA expression using RNA sequencing (RNA-seq).

## Materials and methods

This study was conducted at the Division of Neurology, Department of Brain and Neurosciences, Faculty of Medicine, Tottori University, Yonago, Japan.

Cell culture and microscopic imaging

We previously generated a mouse model of ALS referred to as the DF7 mouse line expressing human mtSOD1-L126delTT with a FLAG sequence at the C terminus [14] and abbreviated here as mtSOD1. DF7 cells, mesenchymal stem cells (A1) (MSCs) obtained from DF7 mice, have been described previously [15]. DF7 cells, along with the FLAG sequence, facilitate the evaluation of SOD1-interacting proteins in a culture system [15,16]. We also used KUM10, an MSC cell line derived from wild-type C57BL/6 mice [17], in these studies.

Primary antibodies anti-Cu/Zn SOD (Enzo Life Science, USA) and anti-FLAG M2 (Merck, Germany), and secondary antibodies Alexa Fluor 647 and Alexa Fluor 488 (Cell Signaling Technology, USA) were used in this study. The anti-SOD1 antibody recognizes human SOD1 as well as mouse Sod1.

Immunoprecipitation (IP) and western blotting

DF7 cells were cultured until they reached approximately 90% confluency, and then protein extraction and fractionation were performed using a nuclear/cytosolic fractionation kit (Cell Biolabs, USA). Five micrograms of anti-FLAG M2 antibody were added to precleared Pierce protein A/G magnetic beads (Thermo Fisher Scientific, USA), and protein extracts of the cytoplasmic or nuclear fraction were added. Immunoprecipitation (IP) was performed according to a magnetic bead-based protocol (Thermo Fisher Scientific). For western blotting, anti-Cu/Zn SOD, anti-FLAG M2, anti-α-tubulin (T9026, Merck), and anti-lamin A/C (Cloud-Clone Corp, USA) were used as primary antibodies. ECL Prime anti-mouse-IgG and ECL Prime anti-rabbit-IgG (GE Healthcare, USA) were used as secondary antibodies.

Data-independent acquisition (DIA) proteomic analyses

Data-independent acquisition (DIA) proteomics is a global mass spectrometry-based proteomics approach characterized by broad protein coverage, high reproducibility, and high accuracy [18]. Nuclear and cytosolic proteins were fractionated using the aforementioned protocol with scale-up modifications, and 500 µg of total protein was obtained from both fractions. IP was performed using 5 µg of anti-FLAG M2 antibody and monoclonal mouse IgG (MOPC 21 clone, Sigma-Aldrich, USA) as the negative control for the nuclear fraction. DIA protein analysis was accomplished by Kazusa Genome Technologies (Chiba, Japan), and the acquired data were analyzed using Scaffold DIA software (Proteome Software, Inc., USA) (https://www.proteomesoftware.com/products/scaffold-dia). For the analysis, only protein/peptide data with both a peptide false discovery rate (FDR) and protein FDR less than 1% and three or more unique peptides were included. To obtain mtSOD1 binding proteins, log2 (fold change, FC) was calculated by taking the log2 conversion of the value ratio (FLAG/control) in the nuclear fraction. Proteins in FLAG samples with log2 FC ≥ 2 and those with missing values only in the control IP were used for the Metascape gene ontology (GO) analysis (v3.5 20230501) [19].

Chromatin IP sequencing (ChIP-seq) and data analysis

DF7 cells (1.5 × 107) were used for the chromatin IP sequencing (ChIP-seq) analysis. The cells were crosslinked with 1% formaldehyde and fragmented using a Covaris M220 ultrasonicator (Covaris, USA). Protein-DNA complexes were immunoprecipitated using an anti-FLAG M2 antibody. The protein-DNA complexes were then de-crosslinked, and DNA was purified using a MinElute PCR purification kit (Qiagen, USA). Sequencing libraries were prepared following ChIP, and input DNA was obtained through the standard consecutive enzymatic steps of end-polishing, dA-addition, and adaptor ligation using a NEBNext Ultra Ⅱ DNA library prep kit for Illumina (E7645, New England BioLabs, USA). These and subsequent steps were performed by Kazusa Genome Technologies. After the final PCR amplification, the resulting DNA libraries were quantified and sequenced on a NextSeq 500 (75 base pair reads, single end, Illumina, USA).

Primary data were analyzed using bcl2fastq ver. 2.20 (Illumina), and FASTQ files were generated from the obtained reads. Trimmomatic [20] was used to exclude sequences with less than 36 bp from the FASTQ format data, and the remaining sequences were mapped against the reference mouse genome (mm10) using Bowtie2 (ver. 2.4.5) [21]. Peak calling and a motif search were performed using HOMER (ver. 4.7) [22]. Motifs known to bind transcription factors were used to search for target genes within ± 5 kbp of the transcription start site (TSS) in the mouse (mm9) genome using ChIP-Atlas [23].

To obtain cell- and tissue-specific gene expression conditions, we referred to the Pattern Gene Database (PaGenBase) [24], which provides information on gene expression patterns based on serial gene expression profiles obtained under multiple physiological conditions. To predict transcription factor regulation, we employed Transcription Regulatory Relationship Unraveled Sentence-based Text (TRRUST) mining, which is a manually curated database of human and mouse transcriptional regulatory networks [25].

RNA interference (RNAi) and RNA sequencing (RNA-seq)

Two RNA sequences were synthesized for RNAi. The first, panSOD1_siRNA (5′-CACUGGUGGUCCAUGAAAA-3′, Nippon Gene, Japan), silences mtSOD1 and mouse Sod1, while mtSOD1_siRNA (5′- GUGGAAAGACUACAAAGACGAdTdT -3′) only silences transgenic mtSOD1. For negative controls, we used a universal negative control RNA sequence (Nippon Gene) that showed no homology with any eukaryotic gene sequence (the sequence is not publicized). The ScreenFect siRNA (Fujifilm, Japan) system was used for RNAi.

DF7 cells (2.0 × 105) were incubated in 2200 µL of RNA-containing medium per well of a six-well plate at 37°C and 5% CO₂ for 48 h. After the silencing procedure, cells were collected with 0.25% trypsin/ethylenediaminetetracetic acid (EDTA), and RNA extraction was performed using NucleoSpin RNA plus XS (Takara Bio, Japan). The purified RNA was assessed by Nanodrop (Thermo Fisher Scientific) measurement. Library preparation and subsequent steps were performed by Veritas Genetics, Inc. (MA, USA). The NEBNext Ultra II RNA Library Prep Kit for Illumina (New England Biolabs) was used as the library preparation kit. Twenty million paired-end (150-bp) reads were sequenced on a Novaseq 6000 (Illumina, USA). RNA-seq Fastq data were quality-checked and analyzed using RaNA-seq [26]. Read count data were analyzed and plotted in integrated Differential Expression and Pathway (iDEP 0.96) [27].

## Results

Immunoprecipitation (IP) nuclear proteomic analysis

**Figure 1:**
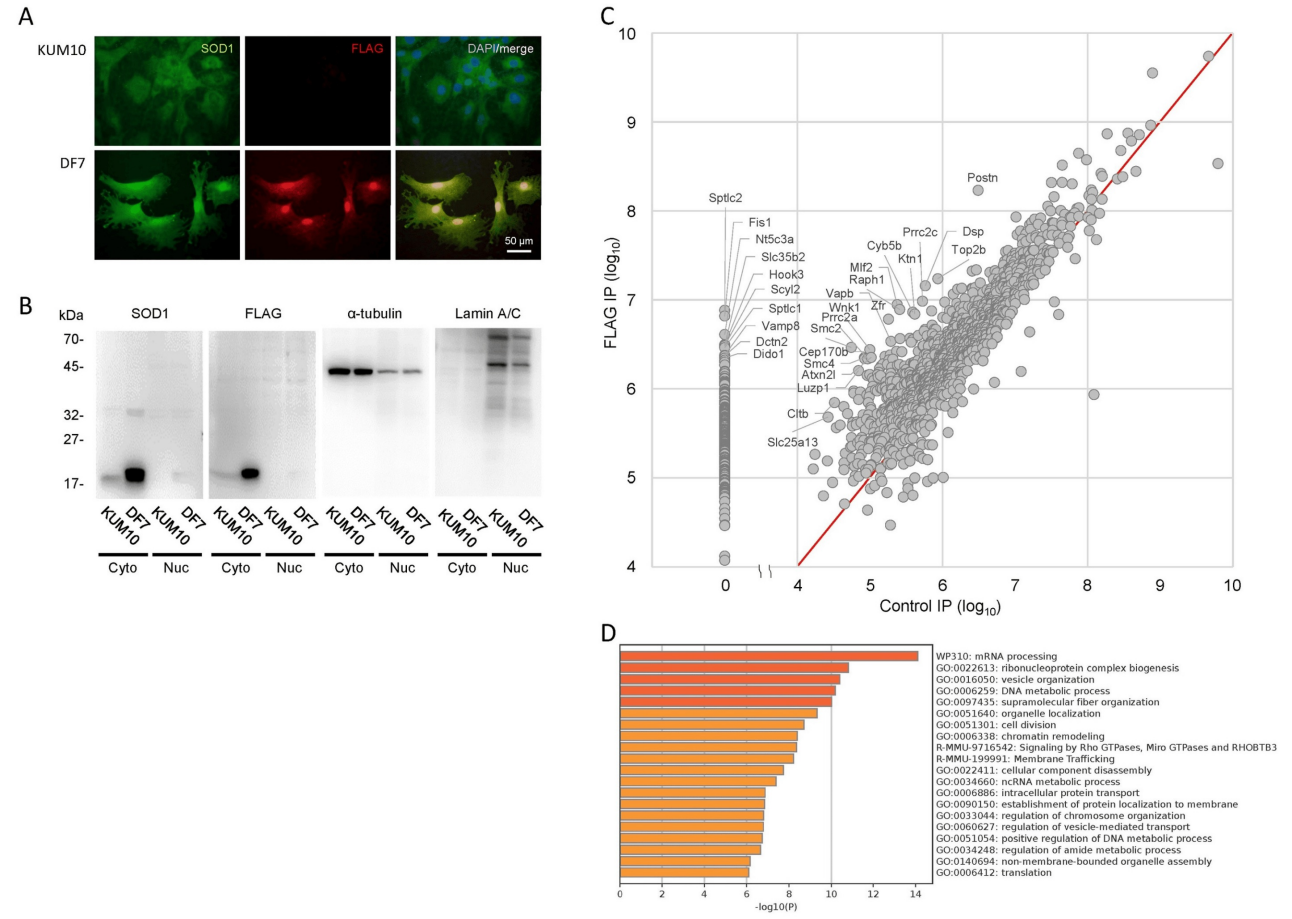
mtSOD1 interacts with proteins in the nucleus (A) SOD1 localization in KUM10 and DF7 cells via immunocytochemistry. Merged images of SOD1 (green) and nuclei (blue) staining were obtained under a BZ-8000 fluorescent microscope. (B) Ten micrograms (20 μg in the DF7 nuclear fraction) of total protein were separated and detected using anti-Cu/Zn SOD1, anti-FLAG M2, anti-α-tubulin, and anti-lamin A/C antibodies. α-Tubulin was detected in all fractions, whereas lamin A/C was only detected in the nuclear fractions of KUM10 and DF7, as it was present in the inner nuclear membrane. The estimated molecular weights of each protein are SOD1 (human and mouse), 16.8 kDa; α-tubulin, 50 kDa; and lamin A/C, 72 kDa and 63 kDa. Cyto: cytosol fraction, Nuc: nuclear fraction. (C) The values for categories (a)–(d) proteins were plotted. The top 10 highest values of category (a) proteins and proteins with log_2_ FC > 4 in category (b) (together, 29 proteins) are indicated with gene symbols. (D) The enrichment GO analysis was performed using proteins in categories (a) and (b). GO: gene ontology; mtSOD1: Cu/Zn superoxide dismutase mutation

DF7, but not KUM10, cells were found positive for FLAG and mtSOD1, indicating the nuclear localization of mtSOD1 in DF7 specifically (Figure 1A). In the western blots of both cytoplasmic and nuclear fractions (Figure 1B), immunoreactivity against anti-SOD1 antibodies was strongly detected in the cytoplasm of both KUM10 and DF7 cells and weakly detected in the DF7 nuclear fraction.

We identified 1,396 mtSOD1-interacting proteins in cell nuclei via FLAG IP (Appendix 1). Among them, 216 proteins were classified as category (a), as they were identified via FLAG IP in the nucleus but were not detectable when using control IgG (Table 1). All of these proteins were found to have FLAG quantitative values of more than 1.0 × 10^4^. In category (b), 176 proteins exhibited a log_2_ FC > 2 in the FLAG IP compared to the control (Table 2). Category (c) consisted of 991 proteins with log₂ FC values between 2 and -2, while category (d) included 13 proteins with log₂ FC < -2. Data for all categories are plotted in Figure 1C. In the top 20 high-value proteins in category (a), serine palmitoyltransferase, long chain base subunit 1 (Sptlc1), which is a causative gene product in early onset ALS (ALS27) [28], and Sptlc2 (the highest value) are listed. Recently, it has been reported that variations in SPTLC2 are also associated with early-onset ALS and frontotemporal dementia (FTD) [29]. Sptlc1 and 2 form serine palmitoyltransferases with other proteins and are known to be a key enzyme in sphingolipid metabolism [30]. Ataxin 2 (Atxn2), mutations of which are found in ALS13 [31] and spinocerebellar ataxia type 2 (SCA2) [32], was also listed within the category (a). Among the 20 highly specific mtSOD1-binding proteins (log_2_ FC > 4) within category (b) (Table 2), vesicle-associated membrane protein-associated protein B/C (Vapb) has been reported as the causative gene for ALS8 [33], and Atxn2l [34], which is a paralog of Atxn2, were listed.

**Table 1:** Top 20 nuclear mtSOD1 interacting proteins from categories (a) * incalculable, the full list of identified proteins is available upon request. mtSOD1: Cu/Zn superoxide dismutase mutation

Protein	Gene symbol	Molecular weight (kDa)	Accession number	Peptide count	Unique peptide count	Protein group score	FLAG IP value	Control IP value	Log_2_ (FLAG/control)
Serine palmitoyltransferase 2	Sptlc2	63	P97363	10	10	0.999	7.7E+06	0.0E+00	*
Mitochondrial fission 1 protein	Fis1	17	Q9CQ92	5	5	0.992	6.6E+06	0.0E+00	*
Cytosolic 5'-nucleotidase 3A	Nt5c3a	37	Q9D020	6	6	0.999	4.1E+06	0.0E+00	*
Protein hook homolog 3	Hook3	83	Q8BUK6	8	8	0.994	3.1E+06	0.0E+00	*
Adenosine 3'-phospho 5'-phosphosulfate transporter 1	Slc35b2	47	Q91ZN5	4	4	0.986	3.1E+06	0.0E+00	*
SCY1-like protein 2	Scyl2	103	Q8CFE4	7	7	0.998	3.0E+06	0.0E+00	*
Serine palmitoyltransferase 1	Sptlc1	53	O35704	7	7	1.000	2.9E+06	0.0E+00	*
Vesicle-associated membrane protein 8	Vamp8	11	O70404	3	3	0.997	2.4E+06	0.0E+00	*
Dynactin subunit 2	Dctn2	44	Q99KJ8	6	6	0.988	2.3E+06	0.0E+00	*
Death-inducer obliterator 1	Dido1	247	Q8C9B9	6	6	0.994	2.2E+06	0.0E+00	*
DNA topoisomerase 2-alpha	Top2a	173	Q01320	11	6	0.995	2.1E+06	0.0E+00	*
Beta-hexosaminidase subunit beta	Hexb	61	P20060	3	3	0.995	2.0E+06	0.0E+00	*
Centrosomal protein of 170 kDa	Cep170	175	Q6A065	8	8	0.999	1.9E+06	0.0E+00	*
DnaJ homolog subfamily A member 1	Dnaja1	45	P63037	7	7	0.999	1.6E+06	0.0E+00	*
Sister chromatid cohesion protein PDS5 homolog B	Pds5b	164	Q4VA53	8	8	0.999	1.5E+06	0.0E+00	*
Tripeptidyl-peptidase 2	Tpp2	140	Q64514	4	4	0.991	1.5E+06	0.0E+00	*
NF-kappa-B-repressing factor	Nkrf	78	Q8BY02	5	5	0.986	1.4E+06	0.0E+00	*
Serine/threonine-protein kinase MARK2	Mark2	86	Q05512	7	4	0.969	1.3E+06	0.0E+00	*
E3 ubiquitin-protein ligase RING2	Rnf2	38	Q9CQJ4	4	4	0.999	1.3E+06	0.0E+00	*
Ribosomal RNA processing protein 1 homolog B	Rrp1b	81	Q91YK2	3	3	0.981	1.2E+06	0.0E+00	*

**Table 2:** Top 20 nuclear mtSOD1 interacting proteins from categories (b) The full list of identified proteins is available upon request. mtSOD1: Cu/Zn superoxide dismutase mutation

Protein	Gene symbol	Molecular weight (kDa)	Accession number	Peptide count	Unique peptide count	Protein group score	FLAG IP value	Control IP value	Log_2_ (FLAG/control)
Periostin	Postn	93	Q62009	11	11	1.000	1.7E+08	3.1E+06	55.269
Structural maintenance of chromosomes protein 2	Smc2	134	Q8CG48	21	21	1.000	2.9E+06	5.5E+04	52.796
Myeloid leukemia factor 2	Mlf2	28	Q99KX1	4	4	0.999	8.9E+06	2.4E+05	37.325
Zinc finger RNA-binding protein	Zfr	117	O88532	7	7	0.997	6.1E+06	1.8E+05	33.717
Ras association (RalGDS/AF-6) and pleckstrin homology domains 1	Raph1	137	F2Z3U3	8	8	0.995	7.8E+06	2.6E+05	30.351
Serine/threonine-protein kinase WNK1	Wnk1	251	P83741	5	5	0.992	2.8E+06	1.0E+05	27.723
Structural maintenance of chromosomes protein 4	Smc4	147	Q8CG47	12	12	0.998	2.2E+06	8.4E+04	26.696
Centrosomal protein of 170 kDa protein B	Cep170b	171	Q80U49	9	9	0.998	2.2E+06	8.5E+04	25.768
Desmoplakin	Dsp	333	E9Q557	17	16	0.997	1.4E+07	5.8E+05	24.933
Protein PRRC2A	Prrc2a	229	Q7TSC1	18	17	0.998	2.2E+06	9.3E+04	23.364
Leucine zipper protein 1	Luzp1	119	Q8R4U7	16	16	0.995	1.6E+06	7.0E+04	23.000
Ataxin-2-like protein	Atxn2l	111	Q7TQH0	13	13	0.999	2.3E+06	1.0E+05	21.730
Clathrin light chain B	Cltb	25	Q6IRU5	6	6	0.988	7.0E+05	3.2E+04	21.626
DNA topoisomerase 2-beta	Top2b	182	Q64511	26	21	0.998	1.7E+07	8.6E+05	20.118
Cytochrome b5 type B	Cyb5b	16	Q9CQX2	5	5	0.997	7.3E+06	3.9E+05	18.748
Protein PRRC2C	Prrc2c	311	Q3TLH4	32	31	1.000	9.7E+06	5.3E+05	18.248
Calcium-binding mitochondrial carrier protein Aralar2	Slc25a13	74	Q9QXX4	7	4	0.985	4.8E+05	2.6E+04	18.191
Vesicle-associated membrane protein-associated protein B	Vapb	27	Q9QY76	6	5	0.999	3.4E+06	1.9E+05	17.653
Kinectin	Ktn1	153	Q61595	22	22	1.000	6.9E+06	4.2E+05	16.712
NADH dehydrogenase [ubiquinone] iron-sulfur protein 4, mitochondrial	Ndufs4	20	Q9CXZ1	3	3	0.995	9.5E+05	6.0E+04	15.835

We determined that the 392 proteins exhibited highly specific interactions with mtSOD1 in nuclei (categories (a) and (b)). These proteins were subjected to Metascape GO analysis (Figure 1D), which revealed that the mtSOD1-interacting proteins in nuclei were predominantly associated with "mRNA processing” (*p* < 10^-14^), followed by “ribonucleoprotein complex biogenesis” (*p* < 10^-10^), “vesicle organization” (*p* < 10^-10^), and “DNA metabolic process” (*p* < 10^-10^). Proteins obtained from FLAG IP in the cytosolic fraction are listed in Table 3.

**Table 3:** Top 20 mtSOD1 interacting proteins in the cytosol The full list of identified proteins is available upon request. mtSOD1: Cu/Zn superoxide dismutase mutation

Protein	Gene symbol	Molecular weight (kDa)	Accession number	Peptide count	Unique peptide count	Protein group score	FLAG nucleus IP value	FLAG cytosol IP value	Log_2_ (nucleus/cytosol)
Vimentin	Vim	54	P20152	46	42	0.999951	5.50E+09	8.90E+09	-0.703
Myosin-9	Myh9	226	Q8VDD5	111	94	0.999975	3.60E+09	2.90E+09	0.295
AHNAK nucleoprotein (desmoyokin)	Ahnak	604	E9Q616	157	157	0.999961	4.80E+08	1.30E+09	-1.450
Elongation factor 1-alpha 1	Eef1a1	50	P10126	11	11	0.999862	1.10E+08	1.20E+09	-3.485
Histone H2B type 1-F/J/L	H2bc15	14	P10853	6	4	0.999275	6.00E+07	1.00E+09	-4.096
Histone H4	H4f16	11	P62806	10	10	0.999786	4.80E+07	9.40E+08	-4.309
Plectin	Plec	534	Q9QXS1	194	192	0.999962	7.20E+08	9.20E+08	-0.361
Prelamin-A/C	Lmna	74	P48678	51	51	0.999951	9.50E+07	7.70E+08	-3.017
Actin, cytoplasmic 1	Actb	42	P60710	19	9	0.999362	6.10E+08	7.40E+08	-0.264
E3 ubiquitin-protein ligase TRIP12	Trip12	224	G5E870	6	6	0.976684	8.60E+05	5.70E+08	-9.356
Albumin	Alb	69	P07724	10	10	0.999223	1.40E+08	4.80E+08	-1.817
Filamin-A	Flna	281	Q8BTM8	79	72	0.999961	7.50E+08	4.50E+08	0.736
Myosin light polypeptide 6	Myl6	17	Q60605	9	9	0.999927	3.80E+08	4.20E+08	-0.171
Fibronectin	Fn1	273	P11276	51	51	0.999967	2.40E+08	3.70E+08	-0.593
Myosin-11	Myh11	227	O08638	13	3	0.977975	4.50E+08	3.40E+08	0.381
Actin, aortic smooth muscle	Acta2	42	P62737	18	9	0.999466	2.50E+08	3.40E+08	-0.450
Peroxiredoxin-1	Prdx1	22	P35700	9	9	0.999802	3.20E+07	3.30E+08	-3.364
Phosphate carrier protein, mitochondrial	Slc25a3	40	Q8VEM8	11	11	0.999389	1.70E+07	2.80E+08	-4.020
ADP/ATP translocase 1	Slc25a4	33	P48962	15	9	0.999824	5.10E+07	2.70E+08	-2.418
LIM domain and actin-binding protein 1	Lima1	84	Q9ERG0	22	22	0.999600	7.40E+07	2.50E+08	-1.769

**Figure 2:**
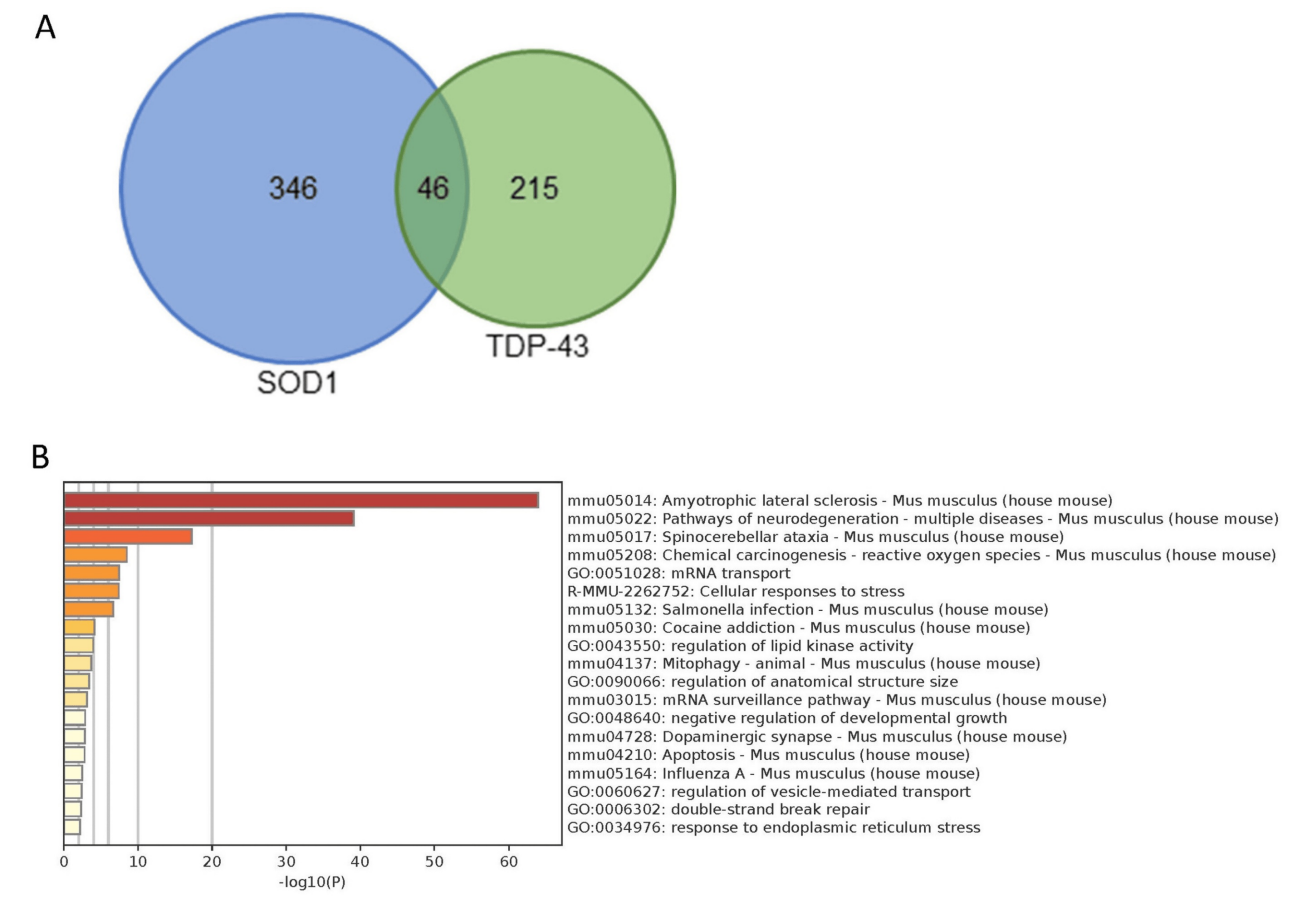
mtSOD1-interacting proteins in nuclei and wild-type TDP-43 interacting proteins (A) A Venn diagram expressing the relationship between mtSOD1-interacting proteins in the nucleus (blue, present experiment) and wild-type TDP-43-interacting proteins (green) is shown. (B) Metascape GO analysis revealed that the most common proteins are known to be related to ALS and neurodegeneration. GO: gene ontology; mtSOD1: Cu/Zn superoxide dismutase mutation; ALS: amyotrophic lateral sclerosis

Freibaum et al. [35] reported a list of proteins that interact with wild-type TAR DNA-binding protein 43 (TDP-43) that were identified by the transfection of expression constructs of FLAG-TDP-43 in mammalian cells. The proteins were not selectively enriched in the nuclear fraction, and a total of 261 TDP-43-interacting proteins were identified. We compared these with our list of 392 proteins (Figure 2A). Forty-six proteins that interact with both mtSOD1 and wild-type TDP-43 are presented in Table 4. From the mtSOD1 analysis, 11.7% of proteins were found to interact with TDP-43, while 17.6% of the wild-type TDP-43-interacting proteins were also found to interact with mtSOD1. Through GO analysis, we found that the overlapping interacting proteins were strongly related to “ALS” and “pathways of neurodegeneration” (Figure 2B).

**Table 4:** mtSOD1-interacting proteins in nuclei and wild-type TDP-43 interacting proteins The 46 proteins in common to both mtSOD1-interacting proteins in the nucleus and wild-type TDP-43-interacting proteins are presented in alphabetical order. mtSOD1: Cu/Zn superoxide dismutase mutation

Gene product
Ago2, Atxn2l, Ddx5, Ddx6, Ddx21, Ddx50, Dhx30, Dhx36, Dsp, Eif3a, Eif4g3, Fubp3, Gnl3, Hnrnpa3, Hnrnpr, Hnrnpu, Ilf3, Jup, Map1b, Map4, Mtdh, Myh9, Nkrf, Nono, Nop2, Nufip2, Nxf1, Pgam5, Prpf3, Prpf19, Prrc2a, Prrc2c, Rpl4, Rpl6, Rpl10a, Rpl32, Rrbp1, Sart1, Serbp1, Sfpq, Srrt, Ssb, Xrn1, Zc3h11a, Zc3hav1, Zfr

Chromatin IP sequencing (ChIP-seq) analysis

**Figure 3:**
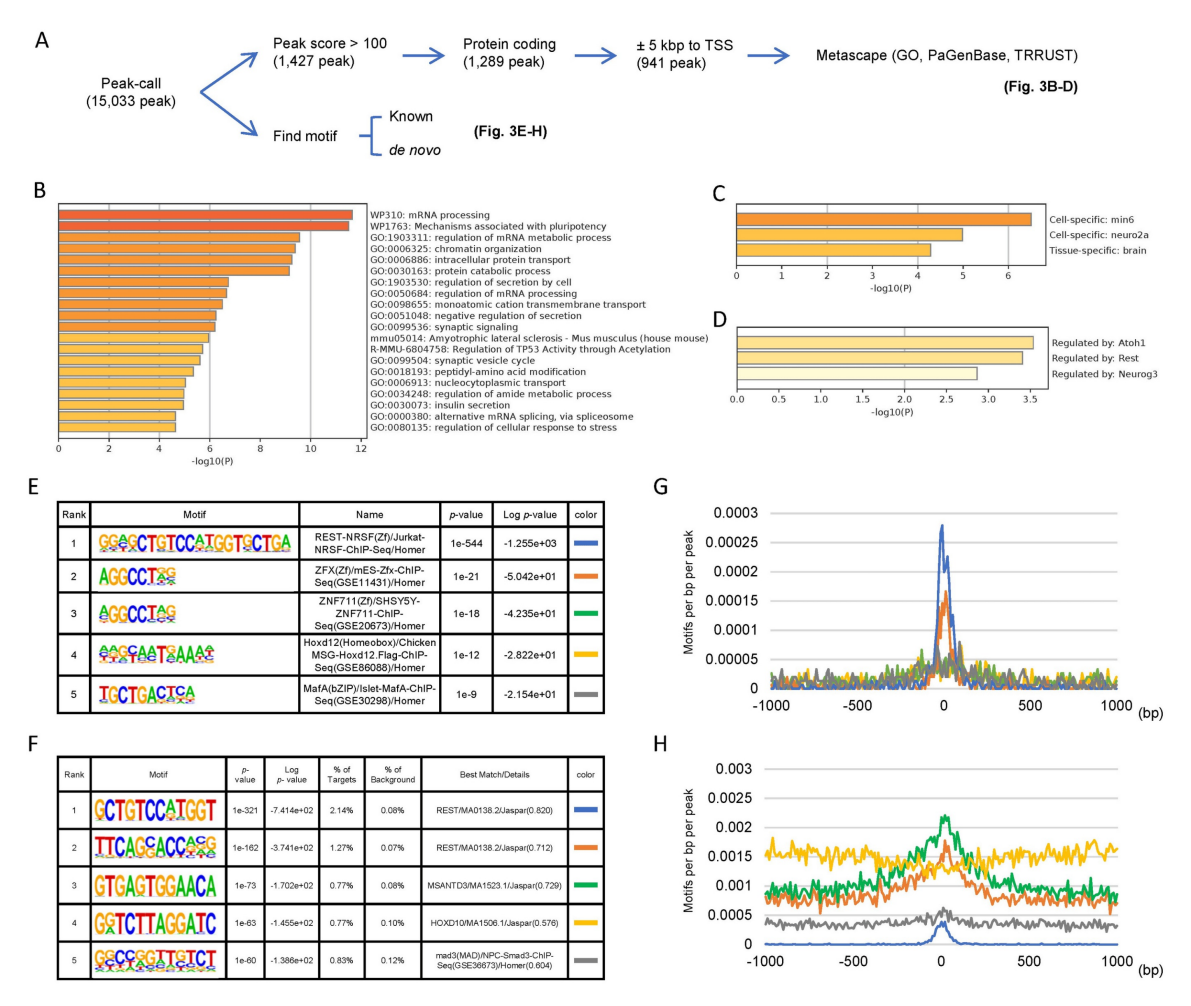
ChIP-seq analysis showing mtSOD1 interactions with DNA (A) Obtained HOMER peaks with a peak score of 100 or more and located within 5 kbp of TSS were used as the input (941 genes) (top). Using the same peak data, known and *de novo* motifs were identified, and their target genes were identified using the ChIP-Atlas database (bottom). (B-D) Metascape GO (B), PaGenBase (C), and TRRUST (D) analyses were performed. (E and F) The top five known (E) and *de novo* (F) motifs were determined. The top known motif corresponded to the *Rest* gene, and the top 1 and 2 *de novo* motifs also matched best with *Rest*. (G and H) Distribution of the top five known (G) and *de novo* (H) motifs within 1 kbp of the peak center are shown. The top 1 and 2 *de novo* motifs and top 1–3 known motifs preferentially interact with DNA regions adjacent to the TSS. GO: gene ontology; mtSOD1: Cu/Zn superoxide dismutase mutation; PaGenBase: Pattern Gene Database; TRRUST: Transcription Regulatory Relationship Unraveled by Sentence-based Text; ChIP-seq: chromatin IP sequencing

A total of 153,360,172 reads were obtained from the input samples, and 250,770,199 reads were obtained from the DF7 ChIP sample (Appendix 1). Peak calling was performed after a quality check of the sequence information. The peaks with scores reaching 100 or more and ± 5 kbp from the TSS (941 peaks, 926 genes) were included in the Metascape GO, PaGenBase, and TRRUST analyses (Figure 3A). GO analysis revealed that the most enriched ontology was “mRNA processing” (*p* < 10^-11^), followed by “mechanisms associated with pluripotency” (*p* < 10^-11^), “regulation of mRNA metabolic process” (*p* < 10^-9^), “chromatin organization” (*p* < 10^-9^), and “intracellular protein transport” (*p* < 10^-9^) (Figure 3B). Remarkably, the list included “ALS” (Figure 3B). Corresponding genes are listed in Table 5. The PaGenBase analysis revealed cell specificity for min6 (mouse-derived pancreatic islet β-cell lines) and neuro2a and brain tissue specificity (Figure 3C). To predict transcription factor regulation, TRRUST was employed, which revealed that mtSOD1 binding sites regulate protein atonal homolog 1 (*Atoh1*), RE1-silencing transcription factor (*Rest*), and neurogenin-3 (*Nuerog3*) (Figure 3D). *Atoh1* and *Neurog3* belong to the basic helix-loop-helix (bHLH) family of transcription factors. *Atoh1* has been reported to be essential for the formation of spinal cord interneurons and many other cell types, while *Neurog3* has the primary function of activating gene transcription in endocrine progenitor cells. *Rest* is a neuron-restrictive silencer [36], which is described later in detail.

**Table 5:** ALS-related genes in Metascape GO analysis ALS-related genes derived from Metascape GO analysis in Figure 3B. GO: gene ontology; ALS: amyotrophic lateral sclerosis

Gene symbol
Actg1, Atf4, Atp5j, Rb1cc1, Cox6c, Gria2, Grin1, Mapk11, Psma2, Psma3, Rac1, Ranbp2, Atxn2, Sem1, Sod1, Tuba1c, Ulk2, Atg13, Nxf1, Alyref2, Adrm1, Tbk1, Cyc1, Map1, c3a, Atp5g2, Ndufb2, Dctn2, Pfn3, Pom121, Wdr41, Srsf7, Ambra1, Tardbp, Setx, Vcp, Rab5a, Nrbf2

Using the HOMER peak data, we then referred to the ChIP-Atlas database to identify target gene sequences for the identified proteins (Figure 3A), and the top five known (Figure 3E) and *de novo* (Figure 3F) binding motifs were identified. The most relevant motif was *Rest*, followed by two other zinc finger protein-binding motifs: zinc finger protein X-linked (*Zfx*) and zinc finger protein 711 (*Znf711*). The top one and two motifs determined in the *de novo* analysis both matched with *Rest*. Next, we generated histograms of the distributions of both known (Figure 3G) and *de novo* (Figure 3H) motifs within a 1 kbp of peak centers. Among the known motifs, *Rest* and *Zfx* exhibited sharp single peaks. Among the *de novo* motifs, the best-matched *Rest* and Myb/SANT-like DNA-binding domain containing 3 (*Msantd3*) motifs also exhibited pronounced peaks. Overall, the pattern of mtSOD1 binding to DNA sequences was specific. 

RNA sequencing (RNA-seq) expression analysis

**Figure 4:**
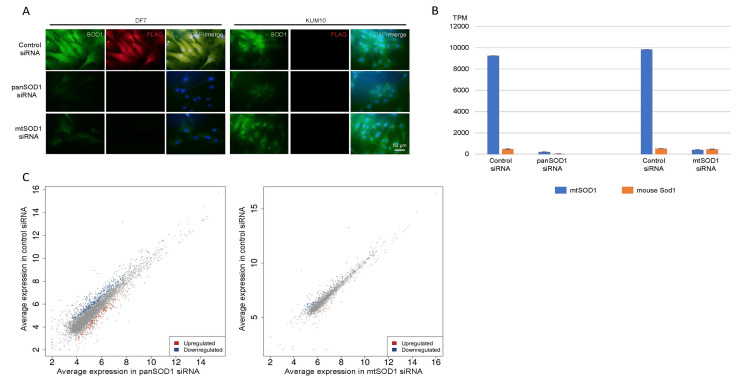
PanSOD1 or mtSOD1 silencing (A) Immunohistochemistry of DF7 and KUM10 at 48h following siRNA treatment. Merged images of FLAG (red), SOD1 (green), and nucleus (blue) staining. Negative control RNA (top), panSOD1 siRNA (middle), and mtSOD1 siRNA (bottom). (B) PanSOD1 siRNA silenced mouse Sod1 and mtSOD1, while mtSOD1 silenced mtSOD1 but not mouse Sod1. (C) Scatter plots of pan-SOD1 (left) and hSOD1-FLAG (right) vs. their respective control siRNAs. mtSOD1: Cu/Zn superoxide dismutase mutation

SOD1 gene silencing was successfully performed (Figures 4A-4C). Both mtSOD1 and mouse Sod1 were silenced via panSOD1 siRNA, while only mtSOD1 was knocked down using mtSOD1 siRNA (Figures 4A, 4B). As the silencing technique reduces both nuclear and cytoplasmic expressions of mtSOD1 and mouse Sod1, and a large fraction of mtSOD1 and mouse Sod1 is distributed in the cytoplasm, it is not possible to separately evaluate the effects of nuclear and cytosolic fractions of mtSOD1 and mouse Sod1 on mRNA expression. The two silencing experiments were performed in an independent manner. RNA-seq data were normalized to convert the data to counts per million (CPM) and then analyzed using iDEP. Differentially expressed genes (DEGs) with log_2_ FCs ≥ 1.5 and adjusted *p-values* < 0.1 were used for the following analysis.

**Figure 5:**
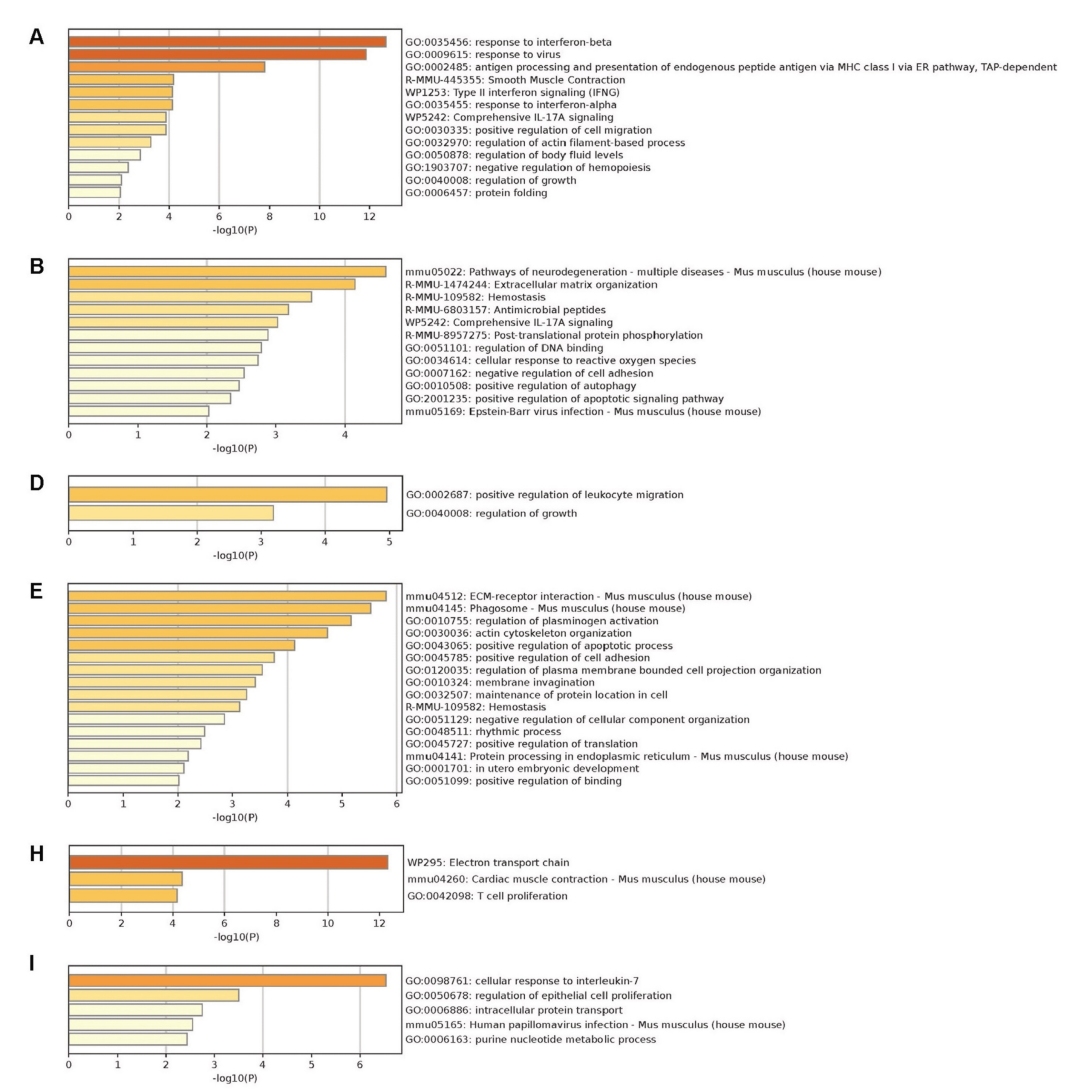
DEGs determined by panSOD1 or mtSOD1 silencing Metascape GO analyses of genes are categorized in columns A, B, D, E, H, and I (Table 6). Highly significant changes (p < 10⁻¹²) were observed in "response to interferon beta" (A) and "electron transport chain" (H). GO: gene ontology; mtSOD1: Cu/Zn superoxide dismutase mutation; DEGs: differentially expressed genes

To further analyze silencing panSOD1 and mtSOD1 or mtSOD1 alone, DEGs identified in both experiments were displayed in nine matrices; for example, corresponding to *Spp1*, the matrix showed no change in panSOD1 and downregulation of mtSOD1 (Table 6). In this table, downregulation indicates that the expression of the genes was downregulated by silencing. Using genes in these nine matrices, Metascape GO analyses were performed (Figures 5A-5I). In the analyses, the most significant finding was that the presence of mouse Sod1 and mtSOD1 was associated with “response to interferon (INF)-β” (*p* < 10^-12^) and “response to virus” (*p* < 10^-10^) (Figure 5A). Because INF-β is secreted in both antiviral and antitumor reactions and is a component of innate immunity [37], these two responses may collectively be referred to as type 1 IFN responses, as we discuss later.

**Table 6:** Genes identified by panSOD1 and mtSOD1 silencing Genes were categorized in a nine-matrix table. Column I shows genes downregulated by panSOD1 silencing and mtSOD1 silencing, while H shows genes downregulated by panSOD1 but showing no changes by mtSOD1 knockdown. Up: upregulated; NC: no change; down: downregulated; mtSOD1: Cu/Zn superoxide dismutase mutation

		Control siRNA v.s. mtSOD1 siRNA		
		Up	NC	Down
Control siRNA v.s. panSOD1 siRNA	Up	A: *Abracl, Acta2, Actg1, Actg2, Apoe, Atf5, Bst2, Ccn5, Cdc34, Col6a3, Csrp1, ENSMUSG00000095742, Fkbp11, Flna, Foxs1, Gadd45g, Gdf15, Gm12895, H2ac19, H2-D1, H2-K1, Hspb6, Ifi204, Ifit1, Ifit3, Ifit3b, Igtp, Irgm1, Isg15, Itga5, Itpripl2, Kdelr3, Lcn2, Map1lc3a, hSOD1-FLAG, Nme2, Oasl2, Pdia5, Pmepa1, Prl2c2, Prl2c3, Rn18s, Rras2, Rtp4,Samd9l, Serpine1, Slc4a2, Slc29a3, Sprr2g ,Stat1, Stat2, Tagln, Tap2, Tgtp2, Tmem140, Tor3a, Tpm2, Trim30a, Usp18*	B: *Adrm1, Ass1, Atox1, Cbr2, Col1a1, Csf1, Cstb, Ddit3, Edf1, Fads3, Fbln2, Ftl1, Gpnmb, Hnrnpab, Ifitm3, Igfbp7, Lcn2, Loxl3, Lpl, Myl6, Myl12a, Niban2, Nid1, Parp3, Pea15a, Ptrh1, Saa3, Scpep1, Slc25a39, Sncg, Sod1, Sparc, Sqstm1, Tagln2, Tapbp, Tgoln1, Tlr2, Tpm4, Tubb4b, Tubb6*	C:
	NC	D: *Btg1, Gas6, Mmp14, Plac8, Rtn4, Timm8b*	E: *Anxa1, Arf3, Atp6v0e, B2m, Bud31, Chmp2a, Ckap4, Cnn2, Col1a2, Col4a1, Cpq, Ctsl, Dap, Dstn, Ecm1, Eef2, Eif3e, Eno1, Fhl2, Fscn1, Gm49909, Grina, H2az1, Hspa5, Id3, Itgb1, Lgals1, Lgals3bp, Maged1, Mbd3, Mt1, Myh9, Myo10, Ndufa2, Nudt4, Pebp1, Pfn1, Pgk1, Pgrmc1, Rpl8, Serpine2, Ssr4, Stk17b, Tceal9, Thbs1, Thbs2, Tmbim4, Tmsb10, Tuba1b*	F: *Spp1*
	Down	G:	H: *Cd24a, Cd164, Cxcl12, Eid1, ENSMUSG0000009504, F3, Gja1, Mgp, mt-Atp6, mt-Co1, mt-Co3, mt-Cytb, mt-Nd4, mt-Nd5, mt-Nd6, Nrn1, Scd2*	I: *Aldh1a2, Atp6v1a, Ccnd2, Chd4, Gas1, Gm15387, Hmgb1, Hspd1, Itih2, Me1, Mir5125, Mmp13, mt-Atp8, Nsg1, Nutf2-ps1, Ociad1, P4hb, Pam, Sar1a*

## Discussion

Nuclear localization of mtSOD1 in physiological and pathological conditions

Wild-type SOD1 (a 32-kDa homodimer protein) is reported to play a key role in protecting DNA from oxidative damage in the nucleus [6,9,10]. In contrast, mtSOD1 might cause DNA damage and trigger apoptosis by activating p53 [6] or disrupting the architecture of nuclear gems [7]. To date, however, knowledge about mtSOD1 in the nucleus is limited.

Generally, proteins smaller than 40 kDa can passively diffuse into the nucleus through the nuclear pore complex (NPC) [38]. Most molecules larger than 40 kDa rely on NPC-dependent transportation systems that carry protein cargo [38]. Nuclear import of proteins is mediated by importin-family proteins, while exportin-1 (XPO1) mediates the nuclear export of proteins. The export of RNA, in contrast, relies on a ribonucleoprotein complex (mRNP) formed by mRNA molecules and RNA-binding proteins, followed by export via NPC by nuclear RNA export factor 1 (Nxf1)/Ntf2-related export protein 1 (Nxt1) [39]. Our proteomic experiment revealed that mtSOD1 bound to Nxf1.

Apart from proteins with physiological roles and those involved in RNA transport, quality control mechanisms for other proteins in the nucleus are as of yet unclarified [40,41]. They are, however, likely to involve proteins exported to the cytosol. Hirayama et al. [40] reported using human cell lines to identify ubiquitinated proteins in the nucleus bound to ubiquilin 4 (UBQLN4), desulfylating isopeptidase 1 (DESI1), and XPO1, which were then exported to the cytosol to be processed by proteasomes. Importantly, UBQLN4 is an ALS-associated gene [42], and mtSOD1 was shown to bind with Ubqln4 in our proteomic experiment. A recent study suggested that an NPC-independent vesicular nucleocytoplasmic transport system may be present in eukaryotic cells and that, through this system, unneeded proteins in the nucleus can be processed through the autophagy lysosomal pathway in the cytosol [41]. Therefore, it is highly possible that the stringency of quality control in the nucleus decreases with age in the same way as it does in the cytosol.

Alteration of mRNA processing

To the best of our knowledge, this is the first proteomic study exploring mtSOD1-interacting proteins in the nucleus. We found that ALS-related gene products, Sptlc1, Atxn2, and Vapb (Figure 2D), interacted with mtSOD1. GO analysis revealed that “mRNA processing” and “ribonucleoprotein complex (mRNP) biogenesis” were the predominant functions of enriched proteins (Figure 1E). Further, over 11% of mtSOD1-interacting proteins in the nucleus were revealed to also interact with wild-type TDP-43 (Figure 2). TDP-43 was identified as the major protein characteristic of sporadic ALS and a causative gene of FALS (ALS10). ALS-related mtSOD1 is well known to lack TDP-43 accumulation [43], leading to the consideration that mtSOD1-related FALS pathogenesis is distinct from pathogenesis associated with TDP-43 [44]. Our present findings shed light on a functional connection between SOD1 and TDP-43.

ChIP-seq analysis of mtSOD1-binding elements revealed that the most enriched GO term was again “mRNA processing” (Figure 3B). Li et al. [8] found that wild-type SOD1 mainly binds to DNA in the vicinity of the TSS in a sequence-specific manner, and three of the top four best-matched motifs were those associated with *ZNF502*, *ZNF583* and *ZNF394* [8]. Our experiment also showed that the top three matched motifs were associated with *Rest*, *Zfx*, and *Znf711* (Figure 2A), all zinc finger family proteins, many of which bind DNA, RNA, and proteins. The most specific SOD1 association was with *Rest*, which is a key regulator of neuron specification and maintenance [45]. Previously, Rockowitz et al. [45] revealed using human and mouse embryonic stem cells to identify several ALS-related gene sequences, including those encoding alsin (ALS2), erb-b2 receptor tyrosine kinase 4 (*ERBB4*, ALS19), fused in sarcoma (*FUS*, ALS6), senataxin (*SETX*, ALS4), and *VAPB*, which are specifically enriched with human-specific *REST* binding genes.

The mtSOD1 interactions with nuclear proteins and DNA motifs could contribute to the pathogenesis of ALS via dysregulation of mRNA processing.

Type 1 IFN response

The GO results from the proteomic and ChIP analyses indicated that mtSOD1 would mostly affect “mRNA processing.” Nonetheless, results of the RNA-seq analysis revealed that the most prominent GO results were “response to IFN-β” and “response to virus.” IFN-β is a type 1 IFN that has antiviral activity and is primarily involved in innate immune responses. In the first line of immunity, viral infections are recognized by cytosolic or endosomal sensors called pattern recognition receptors, and this recognition triggers type 1 IFN responses that induce many antiviral IFN-stimulated genes (ISGs) [46]. Remarkably, an estimated 10% of genes in the human genome are potentially regulated by IFNs [47]. Type 1 IFN responses in neurodegeneration have been widely observed not only in ALS but also in Alzheimer’s disease (AD) and Parkinson’s disease (PD) [48]. Indeed, IFN signaling pathways are activated in several lines of mtSOD1 transgenic mice [49,50]. For example, ISGs were found to be increased in the spinal cords of ALS mice at a presymptomatic age and specifically in astrocytes surrounding motor neurons [49]. Constitutive basal activation of type 1 IFN responses has been shown in the glial cells of several ALS model lines [50]. Mitochondria damaged by mtSOD1 have been speculated to release mitochondrial DNA and RNA/DNA hybrids into the cytoplasm, where these molecules activate type 1 IFN responses [50]. Alternatively, mtSOD1 and nucleic acid complexes and/or mtSOD1 and mRNP complexes from the nucleus may induce type 1 IFN responses. Once type 1 IFN responses are activated, neuroinflammation could exacerbate the progression of ALS.

Motor neuron degeneration

The immortalized cells used in this study duplicate within one to two days under our experimental conditions. This timescale sounds reasonable for mtSOD1 to provoke type 1 IFN responses. Because type 1 IFN responses are acute-phase, glial cell populations can contribute greatly to this response by themselves becoming activated. However, considering that mtSOD1 is expressed in every glial cell from the beginning, it is difficult to explain the occurrence of motor neuron death later in life, as well as region-specific degeneration in the anterior spinal cord and motor cortex.

How, then, might mtSOD1 act in neurons? With some exceptions, neurons do not divide throughout their life span; thus, it is conceivable that interactions between mtSOD1 and nuclear proteins and DNA increase with time. These interactions might compromise “mRNA processing” and other cellular functions, based on the results of our experiments. In addition, a decline in protein quality control, defective energy metabolism, and increased oxidative stress might trigger motor neuron degeneration with aging. Through these mechanisms, we hypothesize that once motor neuron degeneration is initiated, hyperexcitable glial cells induce excessive inflammation adjacent to degenerated motor neurons.

Limitations and future directions

Interactions between mtSOD1 and nuclear proteins and DNA might impact non-dividing neurons. Our current experiment, however, could not replicate this impact *in vitro*. This issue must be addressed by experiments with other cell types, such as *in vitro*-differentiated neurons from stem cells.

In contrast, type 1 IFN responses are widely observed not only in neurodegeneration but also in aging and senescence [48]. We cannot yet clarify the distinct roles of astrocytes, microglia, and neurons in this response. These roles should be clarified in future studies.

## Conclusions

Our study revealed that mtSOD1 interacts with nuclear proteins and specific DNA regions, suggesting a potential role in disrupting mRNA processing. ChIP-seq and RNA-seq analyses further demonstrated that mtSOD1 alters gene expression, particularly enhancing type 1 interferon responses. These findings highlight a functional link between mtSOD1 and nuclear processes, which may contribute to motor neuron degeneration in ALS. For the cure for “gain of function” genetic disorders, antisense oligonucleotide therapy is promising, like FALS with mtSOD1. Still in sporadic ALS, interactions between molecules associated with ALS pathogenesis should be examined in detail. Understanding these nuclear interactions provides novel insights into ALS pathogenesis and may open new avenues for targeted therapeutic interventions.

## Appendices

Appendix 1

Availability of Data and Material

For the proteomic data, the accession numbers are PXD039696 for ProteomeXchange and JPST002013 for jPOST. Chromatin immunoprecipitation-sequencing (ChIP-seq) data are available from the DDBJ Sequence Read Archive (DRA) accession numbers: DRR440373-DRR440374 (watanabe_0-0001_Run_0001-0002).
